# Yields and chondrogenic potential of primary synovial mesenchymal stem cells are comparable between rheumatoid arthritis and osteoarthritis patients

**DOI:** 10.1186/s13287-017-0572-8

**Published:** 2017-05-16

**Authors:** Yuji Kohno, Mitsuru Mizuno, Nobutake Ozeki, Hisako Katano, Keiichiro Komori, Shizuka Fujii, Koji Otabe, Masafumi Horie, Hideyuki Koga, Kunikazu Tsuji, Mikio Matsumoto, Haruka Kaneko, Yuji Takazawa, Takeshi Muneta, Ichiro Sekiya

**Affiliations:** 1 Center for Stem Cells and Regenerative Medicine, Tokyo Medical and Dental University, 1-5-45 Yushima, Bunkyo-ku, Tokyo, 113-8510 Japan; 2 Department of Joint Surgery and Sports Medicine, Tokyo Medical and Dental University, 1-5-45 Yushima, Bunkyo-ku, Tokyo, 113-8510 Japan; 3 Department of Cartilage Regeneration, Tokyo Medical and Dental University, 1-5-45 Yushima, Bunkyo-ku, Tokyo, 113-8510 Japan; 4 Department of Orthopaedic Surgery, Juntendo University Hospital, 3-1-3 Hongo, Bunkyo-ku, Tokyo, 113-8431 Japan; 5 National Hospital Organization Disaster Medical Center, 3256 Midoricho, Tachikawa-shi, Tokyo, 190-0014 Japan

**Keywords:** Synovial mesenchymal stem cells, Rheumatoid arthritis, Osteoarthritis, Cell yields, Chondrogenic potential

## Abstract

**Background:**

Mesenchymal stem cells derived from the synovial membrane (synovial MSCs) are a candidate cell source for regenerative medicine of cartilage and menisci due to their high chondrogenic ability. Regenerative medicine can be expected for RA patients with the inflammation well-controlled as well as OA patients and transplantation of synovial MSCs would also be a possible therapeutic treatment. Some properties of synovial MSCs vary dependent on the diseases patients have, and whether or not the pathological condition of RA affects the chondrogenesis of synovial MSCs remains controversial. The purpose of this study was to compare the properties of primary synovial MSCs between RA and OA patients.

**Methods:**

Human synovial tissue was harvested during total knee arthroplasty from the knee joints of eight patients with RA and OA respectively. Synovial nucleated cells were cultured for 14 days. Total cell yields, surface markers, and differentiation potentials were analyzed for primary synovial MSCs.

**Results:**

Nucleated cell number per 1 mg synovium was 8.4 ± 3.9 thousand in RA and 8.0 ± 0.9 thousand in OA. Total cell number after 14-day culture/1 mg synovium was 0.7 ± 0.4 million in RA and 0.5 ± 0.3 million in OA, showing no significant difference between in RA and OA. Cells after 14-day culture were mostly positive for CD44, CD73, CD90, CD105, negative for CD45 both in RA and OA. There was no significant difference for the cartilage pellet weight and sGAG content per pellet between in RA and OA. Both oil red O-positive colony rate and alizarin red-positive colony rate were similar in RA and OA.

**Conclusions:**

Yields, surface markers and chondrogenic potential of primary synovial MSCs in RA were comparable to those in OA. Synovium derived from RA patients can be the cell source of MSCs for cartilage and meniscus regeneration.

**Electronic supplementary material:**

The online version of this article (doi:10.1186/s13287-017-0572-8) contains supplementary material, which is available to authorized users.

## Background

Mesenchymal stem cells derived from synovial membrane (synovial MSCs) are a candidate cell source for regenerative medicine of cartilage [1–3] and meniscus [4–6] due to their great chondrogenic ability [7, 8]. In clinical practice, the transplantation of autologous synovial MSCs for ten patients with a symptomatic single cartilage lesion in the knee joints was shown to be effective in terms of magnetic resonance imaging (MRI) and patient-oriented outcome scoring [9]. Clinical studies to examine whether the transplantation of autologous primary synovial MSCs regenerate cartilage and meniscus in the knee are currently ongoing. Indications for the use of synovial MSCs in regenerative medicine are extending to the treatment of osteoarthritis (OA).

Rheumatoid arthritis (RA) is an autoimmune disease that causes inflammation in the joints and is accompanied by progressive joint destruction [10]. Significant therapeutic advances have been made in biopharmaceuticals to control inflammation in the joints of RA patients [11]. Where inflammation can be controlled, regenerative medicine, using synovial MSC transplantation, may have the potential to reverse joint damage in these patients.

Some properties of synovial MSCs may vary depending on diseases etiology. We previously reported that the nucleated cell numbers per tissue weight in the synovium of elderly OA patients was higher than that of young patients with anterior cruciate ligament injury [12], which resulted in the different yields of primary synovial MSCs. However, there have been no reports comparing nucleated cell numbers per tissue weight in the synovium and yields of primary synovial MSCs between RA and OA patients. Furthermore, it remains controversial whether or not the pathological condition of RA can affect chondrogenesis of a patient’s synovial MSCs. The purpose of this study was to compare the properties of primary synovial MSCs between RA and OA patients.

## Methods

### Synovial MSCs

**Fig. 1:**
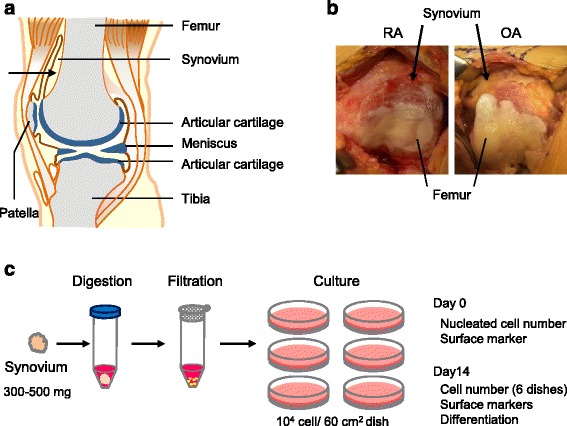
The study scheme. **a** Synovium harvest site. Synovium was collected from the bone side in the suprapatellar pouch (indicated with *arrow*). **b** Representative pictures for synovium in RA and OA patients. **c** Procedures. Synovial tissues (300–500 g) from RA and OA patients were minced, digested, and filtered. One thousand nucleated cells were plated on six dishes (60 cm^2^) and cultured for 14 days. Cells before and after culture were analyzed

This study was approved by local institutional review boards (the Medical Research Ethics Committee of Tokyo Medical and Dental University and the Hospital Ethics Committee of Juntendo University Hospital), and informed consent was obtained from all study subjects. Human synovial tissue was harvested during total knee arthroplasty from knee joints of patients with RA (eight donors) and OA (eight donors) (Fig. 1a, b). The ages of the patients were between 42 and 80 years in RA and between 66 and 81 years in OA (Table 1). The synovial tissues at 300–500 mg from RA (*n* = 5) and OA (*n* = 5) were minced and digested with 3 mg/mL collagenase (Sigma-Aldrich Japan, Tokyo, Japan) in Hanks’ balanced salt solution (Thermo Fisher Scientific, Waltham, MA, USA) at 37 °C for 3 hours (Fig. 1c). The solution was then filtered through a 70 μm nylon filter (Greiner Bio-One GmbH, Frickenhausen, Germany). Digested cells were plated at 10^4^ nucleated cells per 60 cm^2^ dish into six dishes, and cultured in α-minimum essential medium (α-MEM; Thermo Fisher Scientific) supplemented with 1% antibiotic-antimycotic (Thermo Fisher Scientific) and with 10% fetal bovine serum (Thermo Fisher Scientific) without heat-inactivation in a cell culture incubator (Astec, Fukuoka, Japan), set at 37 °C and 5% CO_2_. After being cultured for 14 days at passage 0, total cell yields per dish were obtained, which were harvested with 0.25% trypsin and 1 mM EDTA (Thermo Fisher Scientific) at 37 °C for 5 minutes and were counted with a disposable cell counting plate (One Cell Inc., Shiga, Japan). Then, the expanded cells were used for differentiation assays. To observe colony formation, the dishes were stained with crystal violet.

**Table 1:** Patient demographics

Group	Patient number	Age	Sex	CRP (mg/dl)	ESR (mm)	Medicine		
						PSL (mg/day)	DMARDs other than biologics	Biologics
RA	#1	62	F	0.05	46		MTX	ADA
	#2	67	F	0.49	29		MTX	
	#3	43	F	0.2	26	2	MTX	
	#4	48	F	0.06	39			
	#5	42	F	0.3	15	16	MTX	GLM
	#6	80	F	4.2	71		TCR, SASP, BUC	
	#7	76	M	5.9	111		MTX	
	#8	62	F	0.5	38			
OA	#9	66	F	0.02	10			
	#10	72	F	0.42	28			
	#11	75	F	0.07	14			
	#12	74	F	0.1	40			
	#13	74	F	0.03	9			
	#14	71	F	0.02	14			
	#15	78	F	0.12	18			
	#16	81	F	0.03	18			

Male vs. female was 1:7 in the RA group and 0:8 in the OA group, showing no significant difference (*p* = 0.99 by Mann-Whitney’s *U* test). The mean of age was 60 years old in the RA group, and 74 years old in the OA group: no significant difference between them (*p* = 0.06 by Mann-Whitney’s *U* test)

*CRP* C-reactive protein, *ESR* erythrocyte sedimentation rate, *PSL* prednisolone, *DMARDS* disease-modifying antirheumatic drugs, *MTX* methotrexate, *RA* rheumatoid arthritis, *ADA* adalimumab, *GLM* golimumab, *TCR* tacrolimus,, *SASP* salazosulfapyridine, *BUC* bucillamine, *OA* osteoarthritis

### Flow cytometric analysis

Flow cytometric analyses of synovium-derived cells from RA and OA were performed with digested cells before plating (day 0) and expanded cells cultured for 14 days. The cells from three donors were harvested using a cell dissociation buffer. Cells were suspended in HBSS at a density of 5 × 10^5^ cells/mL and stained for 30 minutes on ice with the antibodies CD11b-PE, CD11c-PE-Cy7, CD14-APC, CD31-FITC, CD44-APC-H7, CD45-FITC, CD73-BV421, CD90-PE, CD105-PerCP-Cy5.5, CD206-FITC, and HLA-DR-APC (Becton, Dickinson and Company; BD, Franklin Lakes, NJ, USA). Flow cytometric analysis of cell surface antigens was performed by a triple-laser FACS Verse™ system (BD).

### Differentiation assay

For chondrogenesis, 250 thousand synovial MSCs were transferred to a 15 ml tube (BD Falcon) and cultured in chondrogenic induction medium containing 10 ng/ml transforming growth factor-β3 (Miltenyi Biotec Japan, Tokyo, Japan) and 500 ng/ml bone morphogenetic protein 2 (BMP-2, Infuse; Medtronic, Minneapolis, MN, USA), which was changed every 3–4 days. After 21 days, the cell pellets were sectioned and stained by safranin O (Wako, Tokyo, Japan). The representative slide for each donor was quantified using a Bern score [13]. These scores were evaluated by two independent observers in a blinded manner and the mean of the score for each donor was shown.

For adipogenesis, synovial MSCs were plated at 100 cells per 60 cm^2^ dish and cultured for 14 days to make cell colonies. The adherent cells were cultured in adipogenic medium supplemented with 100 nM dexamethasone, 0.5 mM isobutyl-methylxanthine (Sigma-Aldrich) and 50 mM indomethacin (Wako) for an additional 14 days, which was changed every 3–4 days. Adipocyte colonies were stained with oil red O staining (Muto Pure Chemicals, Tokyo, Japan) and the same dishes were then stained with crystal violet. Oil red O-positive colony rate was calculated as positive colony number per total colony number.

For calcification, 100 cells were transferred to a 60 cm^2^ dish and cultured for 14 days in culture medium. The adherent cells were cultured in calcification medium containing 50 μg/ml ascorbic acid 2-phosphate (Wako), 10 nM dexamethasone (Wako), and 10 mM β-glycerophosphate (Sigma-Aldrich), which was changed every 3–4 days. After 14 days, calcification was assessed by alizarin red staining (Merck Millipore, Billerica, MA, USA), and the same dishes were then stained with crystal violet. Alizarin red-positive colony rate was calculated as positive colony number per total colony number.

### Immunostaining for type II collagen

Paraffin-embedded sections were deparaffinized, rehydrated, and pretreated with 0.2 mg/ml proteinase K (Dako, Copenhagen, Denmark) in phosphate-buffered saline containing 0.3% Tween-20 for 15 minutes at room temperature. Endogenous peroxidases were quenched using 3% hydrogen peroxidase in methanol for 10 minutes at room temperature. The sections were first incubated with normal horse serum (Vectastain Universal Elite ABC Kit; Vector Laboratories, Burlingame, CA, USA) and then incubated overnight at room temperature with the diluted antibodies (1:200 dilution; Kyowa Pharma Chemical, Toyama, Japan) in a humidified chamber. The sections were then incubated for 30 minutes with biotinylated secondary antibody, followed by 30 minutes incubation with streptavidin–peroxidase complex (Vectastain Universal Elite ABC Kit) at room temperature. The signal was developed as a brown reaction product using peroxidase substrate diaminobenzidine (DAB plus; Dako). Counterstaining was performed with Mayer’s hematoxylin.

### Sulfated glycosaminoglycan (sGAG) content

Pellets were digested in 100 μg/ml papain (Sigma-Aldrich) for 18 hours at 65 °C and dissolved in 200 mM sodium phosphate buffer (Na_2_HPO_4_-NaH_2_PO_4_) containing 100 mM sodium acetate, 5 mM cysteine HCl and 10 mM EDTA-2Na. The sGAG concentration of supernatant was determined by the Blyscan assay (Biocolor Ltd., Newtonabbay, Ireland) according to the manufacturer’s instructions. For each donor, five pellets were digested together and final sGAG content was then divided by five.

### RNA isolation and RTRT-PCR analysis

For chondrogenesis, six pellets for each donor were digested together. For adipogenesis, MSCs were plated at 50 cells/ cm^2^ in 145 cm^2^ plates and precultured for 7 days, then cultured in adipogenic medium for an additional 21 days.

Total RNA was extracted using TRIzol (Thermo Fisher Scientific). cDNA was synthesized with random hexamer primers from total RNA using the Transcriptor High Fidelity cDNA Synthesis kit (Roche Diagnostics, Basel, Switzerland). Reverse transcription (RT) was performed by a 30-minute incubation at 55 °C followed by a 5-minute incubation at 85 °C. Real-time PCR was performed in a LightCycler 480 instrument (Roche Diagnostics). After an initial denaturation step (95 °C for 10 minutes), amplification was performed for 45 cycles (95 °C for 10 seconds, 60 °C for 30 seconds). Relative amounts of mRNA were calculated and standardized as previously described [14–16]. TaqMan gene expression assay kits (Applied Biosystems, Foster City, CA USA) were Hs00153936_m1 for aggrecan, Hs00264051_m1 for type II collagen, Hs00173425_m1 for lipoprotein lipase (LPL), Hs01115513_m1 for peroxisome proliferator activated receptor γ (PPARγ), and the PCR primer of β-actin was as follows: forward 5’-ATTGGCAATGAGCGGTTC-3’, reverse 5’-TGAAGGTAGTTTCGTGGATGC-3’ and universal probe No. 11 (Roche Diagnostics).

### Alkaline phosphatase (ALP) activity

Synovial MSCs were plated at a density of 8 × 10^4^ cells/ well in 12-well plates and precultured for 7 days, then cultured in calcification medium for additional 14 days. After adding 200–300 μl of 0.05% polyoxyethylene (10) octylphenyl ether (Wako) to each well and two rounds of freezing-thawing, the solutions were centrifuged at 15,000 rpm for 15 minutes. ALP activity in the supernatant was measured by a *p*-nitro phenyl-phosphate method using a LabAssay ALP (Wako). In all samples, measurements were performed in duplicate and data were calibrated by protein quantity using a BCA Protein Assay Kit (Thermo Fisher Scientific).”

### Statistical analysis

The results were analyzed using Mann-Whitney’s *U* test with GraphPad Prism 6 (GraphPad Software, La Jolla, CA, USA). *P* values < 0.05 were considered to be significant.

## Results

### Nucleated cell number per synovium weight

**Fig. 2:**
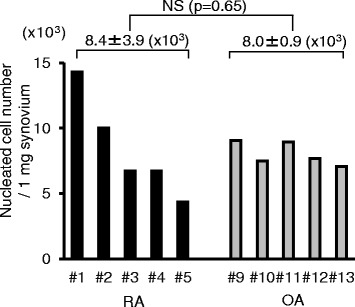
Nucleated cell number per 1 mg synovium. Donor number of RA and OA patients shown. *OA* osteoarthritis, *RA* rheumatoid arthritis

Nucleated cell numbers per 1 mg synovium ranged between 4.4 thousand and 14.3 thousand in RA, and between 7.1 thousand and 9.0 thousand in OA (Fig. 2), showing that the value was distributed more widely in RA than in OA. The average value was 8.4 ± 3.9 thousand in RA and 8.0 ± 0.9 thousand in OA, demonstrating no significant difference between both groups.

### Colony formation and cell morphology

**Fig. 3:**
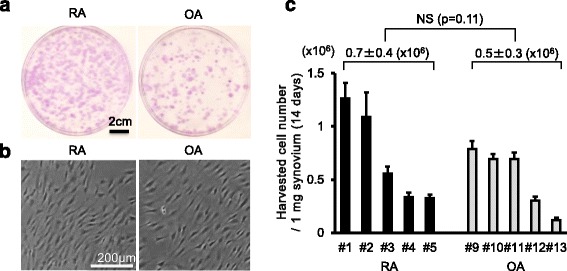
Synovial MSCs from RA and OA 14 days after culture. **a** Representative cell colonies stained with crystal violet. **b** Representative cell morphologies. **c** Harvested cell number per 1 mg synovium after 14 days. Average values with standard deviations are shown (*n* = 6). *OA* osteoarthritis, *RA* rheumatoid arthritis

Fourteen days after 10^4^ nucleated cells were cultured in 60 cm^2^ dishes, similar colony formations were observed in both RA and OA groups (Fig. 3a). Synovial MSCs from OA and RA showed similar spindle-shaped morphologies (Fig. 3b).

### Cell yields

Harvested cell numbers after 14 days of culture ranged between 0.3 million and 1.3 million in RA, and between 0.1 million and 0.8 million in OA (Fig. 3c). The average value was 0.7 ± 0.4 million in RA and 0.5 ± 0.3 million in OA, showing no significant difference between groups.

### Surface markers

**Fig. 4:**
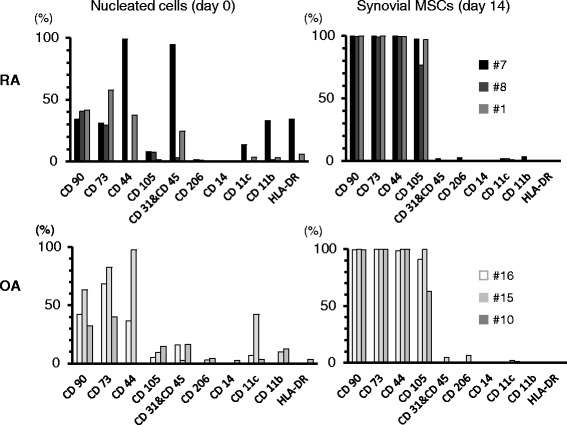
Cell surface markers for nucleated cells and synovial MSCs. Donor number of RA and OA are shown *MSCs* mesenchymal stem cells, *OA* osteoarthritis, *RA* rheumatoid arthritis

Synovial nucleated cells before culture generally expressed hematopoietic markers such as HLA-DR, CD11b, CD11c, CD31 and CD45 at low or moderate levels, and MSC markers such as CD44, CD73, CD90 and CD105 at moderate levels in both RA and OA groups (Fig. 4, Additional file 1: Figure S1 and Figure S2 and Additional file 2: Table S1). Synovial MSCs 14 days after culture were nearly absent for hematopoietic markers and fully expressed MSC markers in both RA and OA groups. No obvious differences could be observed between OA and RA both in total nucleated cells and isolated synovial MSCs.

### Chondrogenesis

**Fig. 5:**
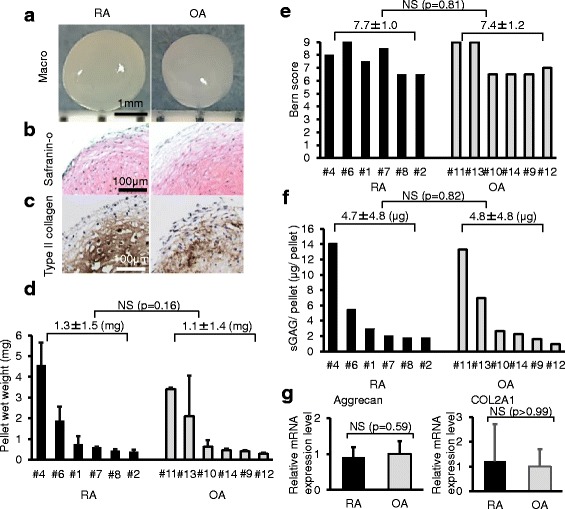
Chondrogenic potential. Synovial MSCs from RA and OA patients were pelleted and cultured in chondrogenic medium for 21 days. **a** Representative macro pictures. **b** Histological sections stained with safranin O. **c** Histological sections immunostained with type II collagen. **d** Wet weight of the pellets. Average values with standard deviations are shown (n = 6). **e** Bern score for the evaluation of safranin O-stained cartilage pellet. **f** sGAG content per pellet. **g** Aggrecan and COL2A1 mRNA expression by real-time RT-PCR analyses. Gene expression fold changes are shown where the expression levels were normalized to OA as 1. Average values with standard deviations are shown (n = 6; RA: # 1, 2, 3, 4, 6, 7; OA: # 9, 10, 11, 12, 13, 14). *OA* osteoarthritis, *RA* rheumatoid arthritis, *sGAG* sulfated glycosaminoglycan

After chondrogenic induction, pellets from RA and OA became spherical, transparent (Fig. 5a), positively stained for sGAG by safranin O (Fig. 5b), and were positive for type II collagen by immunostaining (Fig. 5c), indicating that both cells differentiated into cartilage. Though the wet weight of the cartilage pellets varied among donors, there was no significant difference in pellet weight between RA and OA groups (Fig. 5d). Bern scores, for the evaluation of safranin O-stained cartilage pellets, were comparable between both groups (Fig. 5e). There was no significant difference in sGAG content per pellet between RA and OA groups (Fig. 5f). Real-time RT-PCR analyses showed similar expression levels of aggrecan and COL2A1 for the pellets derived from RA and OA (Fig. 5g).

### Adipogenesis

**Fig. 6:**
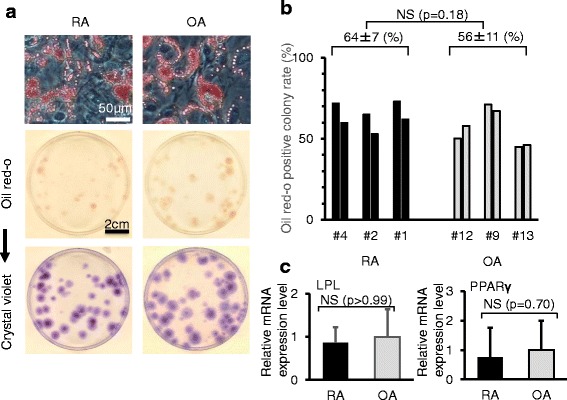
Adipogenic potential. **a** Representative cells and dishes stained with oil red O. The same dishes stained with crystal violet are also shown. **b** Oil red O-positive colony rate. Two dishes were analyzed for each patient. **c** Lipoprotein lipase (*LPL*) and peroxisome proliferator activated receptor γ (*PPARγ*) expression by real-time RT-PCR analyses. Gene expression fold changes are shown when the expression levels were normalized to OA as 1. Average values with standard deviations are shown (n = 3; RA: # 5, 6, 8; OA: # 9, 11, 12). *OA* osteoarthritis, *RA* rheumatoid arthritis

After adipogenic induction, lipid droplets were observed in cells, and approximately half of the total colonies were positively stained with oil red O in RA and OA groups (Fig. 6a). Oil red O-positive colony rates were similar in RA and OA groups (Fig. 6b). Real-time RT-PCR analyses showed similar expression levels of lipoprotein lipase (LPL) and peroxisome proliferator activated receptor γ (PPARγ) for between RA and OA (Fig. 6c).

### Calcification

**Fig. 7:**
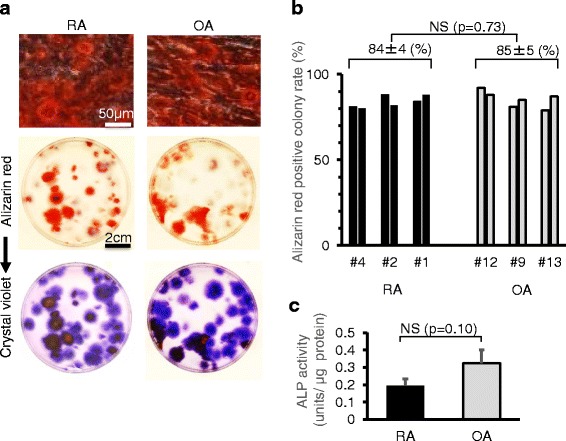
Calcification potential. **a** Representative cells and dishes stained with alizarin red. The same dishes stained with crystal violet are also shown. **b** Alizarin red-positive colony rate. Two dishes were analyzed for each patient. **c** Alkaline phosphatase (*ALP*) activity. Average values with standard deviations are shown (n = 3; RA: # 1, 2, 4; OA: # 9, 12, 13). *OA* osteoarthritis, *RA* rheumatoid arthritis

After calcification induction, most colonies were positively stained with alizarin red in RA and OA groups (Fig. 7a). Alizarin red-positive colony rates were similar in RA and OA groups (Fig. 7b). There was no significant difference in ALP activity between RA and OA (Fig. 7c).

## Discussion

Though the nucleated cell number per synovium weight was similar between RA and OA groups, the distribution was wider in RA than in OA groups. This indicates that synovial tissue was more diverse in RA than in OA from the standpoint of nucleated cell number. It will be interesting to examine the relationship of cell number to histological observations and nucleated cell number per synovium weight after enzyme digestion [17].

The number of cells harvested after 14 days of culture was similar in RA and OA groups. Whereas, we recently demonstrated a relationship between the synovitis score and the total yield of MSCs derived from synovium in a rat carrageenan-induced arthritis model [18]. In the current human study, inflammation was well controlled in RA # 1–5 patients and OA # 9–13 patients when synovium was harvested because their C-reactive protein (CRP) levels were within normal limits. In RA # 1 and # 2 patients, the higher harvested cell numbers were possibly a reflection of past inflammation. Higher harvested cell numbers were also reflected by the higher cell number per synovium weight.

The nucleated cell numbers per synovium weight and harvested cell numbers were relatively low in RA # 3–5 patients. Two of the three patients took prednisolone, which may suggest that glucocorticoid affected these values. We recently observed that harvested synovial MSC numbers after 14 days of culture were extremely low from an OA patient who had glucocorticoid injected into the knee joint 1 week before the synovium was harvested.

Regarding cell surface markers, contrary to our expectation, expression of HLA-DR, CD11b, CD11c, CD14, and CD206, markers for macrophages and monocytes, were relatively low in synovial nucleated cells before culture. However, expression rates for HLA-DR, CD11b, and CD11c were relatively high in RA #7 patient whose CRP was 5.9, which was also high. Expression rates of macrophage and monocyte markers in synovial nucleated cells were possibly a reflection of inflammation when the synovium was harvested. The cells after 14 days of culture were primarily positive for CD44, CD73, CD90, and CD105, and negative for CD45 both in RA and OA groups, which indicated that synovial MSCs acquired properties of MSCs with regard to surface markers.

Regarding CD105, Jones et al. reported that “synovial fibroblastic/stromal cells”, which were negatively selected for CD45 and CD31, expressed CD105 at moderate levels and synovial MSCs, which were expanded by “synovial fibroblastic/stromal cells”, expressed CD105 at high levels in RA patients [19]. In our study, synovial MSCs expressed CD105 at high levels, which was relevant to synovial MSCs by Jones et al. These suggest that synovial MSCs expressed CD105 at high levels irrespective of initial sorting of synovial nucleated cells.

There was no significant difference in cartilage pellet weight between RA and OA groups, which demonstrated that chondrogenic potentials of synovial MSCs between RA and OA patients were similar, as the weight of the pellet is a quantitative indicator of the ability of MSCs to produce chondrogenesis in vitro. According to our previous studies, during the in vitro chondrogenesis of MSCs, the pellet increased in size, weight, and cartilage matrix synthesis. Conversely, the DNA yield per pellet decreased. The amount of DNA in the cells, assessed by prelabeling with 3H-thymidine, was found to be stable during the in vitro chondrogenesis of MSC [20]. These results indicate that the increase in pellet size can be attributed to the production of extracellular matrix, and not the proliferation of the cells.

We also quantified safranin O-stained cartilage pellets using the Bern score to account for uniformity and intensity of matrix staining (0–3 points), cell density/extent of matrix produced (0–3 points), and cellular morphologies (0–3 points), in which 9 points is the maximum score possible [13]. The Bern score was high in most donors irrespective of pellet size. This indicates that the Bern score is likely suitable to evaluate chondrogenic ability on a per cell basis which is then averaged and does not necessarily reflect chondrogenic ability per population of cells. Furthermore, we quantified sGAG content for the pellets. There was no significant difference in the content of sGAG per pellet between RA and OA groups. Similar to our previous report [21], pellet wet weight and sGAG content per pellet were correlated in each donor in both RA and OA groups. Additional experiments by real-time quantitative RT-PCR were performed, and showed similar expression levels of aggrecan and COL2A1 for pellets derived from RA and OA.

Whether the chondrogenic potential of synovial MSCs from RA is similar to that from OA patients is controversial (Table 2). According to previous reports, the chondrogenic potential of passaged synovial MSCs in RA patients was similar to that in OA patients by Skalska et al. [22] and Koizumi et al. [23], contrarily, that in RA patients was inferior to that in OA patients by Jones et al. [19]. Jones et al. collected synovial tissues for biopsy and CRP in donors ranging between 0 and 15 mg/dl, contrarily the other studies, including ours, collected synovial tissues primarily during total knee arthroplasty and CRP in donors ranging only between 0 and 8 mg/dl. The discrepancy in the reported chondrogenic potential of synovial MSCs in RA appears to be due to the different levels of inflammation in the donors [19]. In previous reports, inflammatory cytokines such as interleukin (IL)-1β, tumor necrosis factor alpha (TNFα) and IL-17 inhibited the chondrogenic differentiation of bone marrow MSCs [24, 25].

**Table 2:** Summary of previous and current reports for comparison of synovial MSCs between RA and OA patients

Author	Jones [15]	Skalska [17]	Koizumi [18]	Kohno
Sample number	RA (n = 45) OA (n = 11)	RA (n = 24) OA (n = 18)	RA (n = 13) OA (n = 6)	RA (n = 8) OA (n = 8)
RA synovium collection method	Biopsy with arthroscopy	TKA	TKA, arthroplasty for forefoot, synovectomy for hand	TKA
Passage number for analysis	Passage 3	Passage 4	Passage 2–4	Passage 0
Comparison between RA and OA	Surface epitopes: similar	Surface epitopes: similar	Gene expression: similar	Nucleated cell number: similar
	Chondro: RA < OA	Chondro: similar	In vitro and in vivo	Yields: similar
			Chondro: similar	Surface epitopes: similar
				Chondro: similar
CRP	0–15(mg/dl)	0.5–8 (mg/dl)	0–0.3 (mg/dl)	0–6 (mg/dl)

*RA* rheumatoid arthritis, *OA* osteoarthritis, *TKA* total knee arthroplasty, *Chondro* chondrogenesis, *CRP* C-reactive protein

The addition of overwhelming differentiation stimuli, such as growth factors, could potentially blur the difference between the chondrogenic ability of multiple cell types. Our chondrogenic differentiation medium contained 500 ng BMP-2. We had previously examined the dose effect of BMP-2 on the in vitro chondrogenesis of synovial MSCs and found that cartilage pellets treated with 1000 ng BMP-2 were larger than those treated with 500 ng BMP-2 (data not shown), demonstrating that 500 ng BMP-2 did not show the maximum effect of BMP-2 for the in vitro chondrogenesis of synovial MSCs. These reveal that the in vitro chondrogenesis of synovial MSCs treated with 500 ng BMP-2 is a useful method to compare chondrogenesis of multiple populations of cells [7, 12, 17].

In our study, passage 0 synovial MSCs were analyzed, while in other previous reports, passage 2–4 synovial MSCs were examined (Table 2). Ermis et al. reported that the proportion of synovial cells with trisomy 7 increased strikingly with passage in RA patients [26, 27]. Furthermore, Ben-David et al. revealed that MSCs acquired characteristic large chromosomal aberrations at a similar, or somewhat lower, frequency to that seen in pluripotent stem cells, sometimes within a few passages in culture in *Cell Stem Cell*, 2011 [28]. Moreover, Ben-David et al. proposed that transplantation of MSCs may result in tumor formation, though this was debated between Sensebé et al. [29] and Ben-David et al. [30] in *Cell Stem Cell*, 2012. While it may still be controversial whether chromosomal abnormalities in MSCs might have harmful effects, there are several other reports showing MSCs acquiring chromosomal abnormalities with passaging [31–33]. Therefore, we considered the use of “passage 0” synovial MSCs to be of value in terms of preventing possible risks related to chromosomal abnormalities, regardless of the debate surrounding the severity of those risks.

Though there was no significant difference, the donor age in the RA group was younger than that in the OA group in our study, because the age of patients who undergo TKA is usually younger in RA than in OA. To compare properties of synovial MSCs between RA patients and OA patients, we selected to adjust by knee condition rather than to equalize by donor age. Our ultimate goal is to regenerate cartilage and meniscus with autologous synovial MSCs instead of TKA in both RA and OA patients. We previously collected data on the properties of synovial MSCs derived from OA patients who underwent TKA [12, 17]. Therefore, we chose RA patients who underwent TKA as donors in this study.

Indications for regenerative medicine with primary autologous synovial MSCs are currently extending to OA. Regenerative medicine is also expected to show promise in RA patients with well-controlled inflammation with the transplantation of synovial MSCs as a possible therapeutic treatment. Our results support the future development of joint regenerative medicine capabilities for cartilage and menisci in RA patients as well as OA patients from the standpoint of cell yields, surface markers and chondrogenic potentials.

## Conclusions

We compared properties of primary synovial MSCs between RA and OA patients. Cell yields, surface markers and chondrogenic potentials of synovial MSCs in RA patients were comparable to those in OA patients. Synovium derived from RA patients was shown to be a promising source of MSCs for cartilage and meniscus regeneration.

## Additional files

Additional file 1: Representative flow cytometry diagrams and isotype controls. (ZIP 836 kb)

Additional file 2: Representative raw data for surface markers. Flow cytometric analyses of digested cells before plating (day 0) and expanded cells cultured for 14 days. Donor number of RA and OA patients shown. (DOCX 16 kb)

## Abbreviations

ALP: Alkaline phosphatase
BMP-2: Bone morphogenetic protein 2
CRP: C-reactive protein
MSCs: Mesenchymal stem cells
OA: Osteoarthritis
RA: Rheumatoid arthritis
sGAG: Sulfated glycosaminoglycan
α-MEM: Alpha minimum essential medium
