# Supplementary figures and images for: Yields and chondrogenic potential of primary synovial mesenchymal stem cells are comparable between rheumatoid arthritis and osteoarthritis patients
Source: Stem Cell Res Ther. 2017 May 16;8:115. doi: 10.1186/s13287-017-0572-8 (PMC5434623; doi:10.1186/s13287-017-0572-8)

Supplementary Figure

Flow cytometry diagram

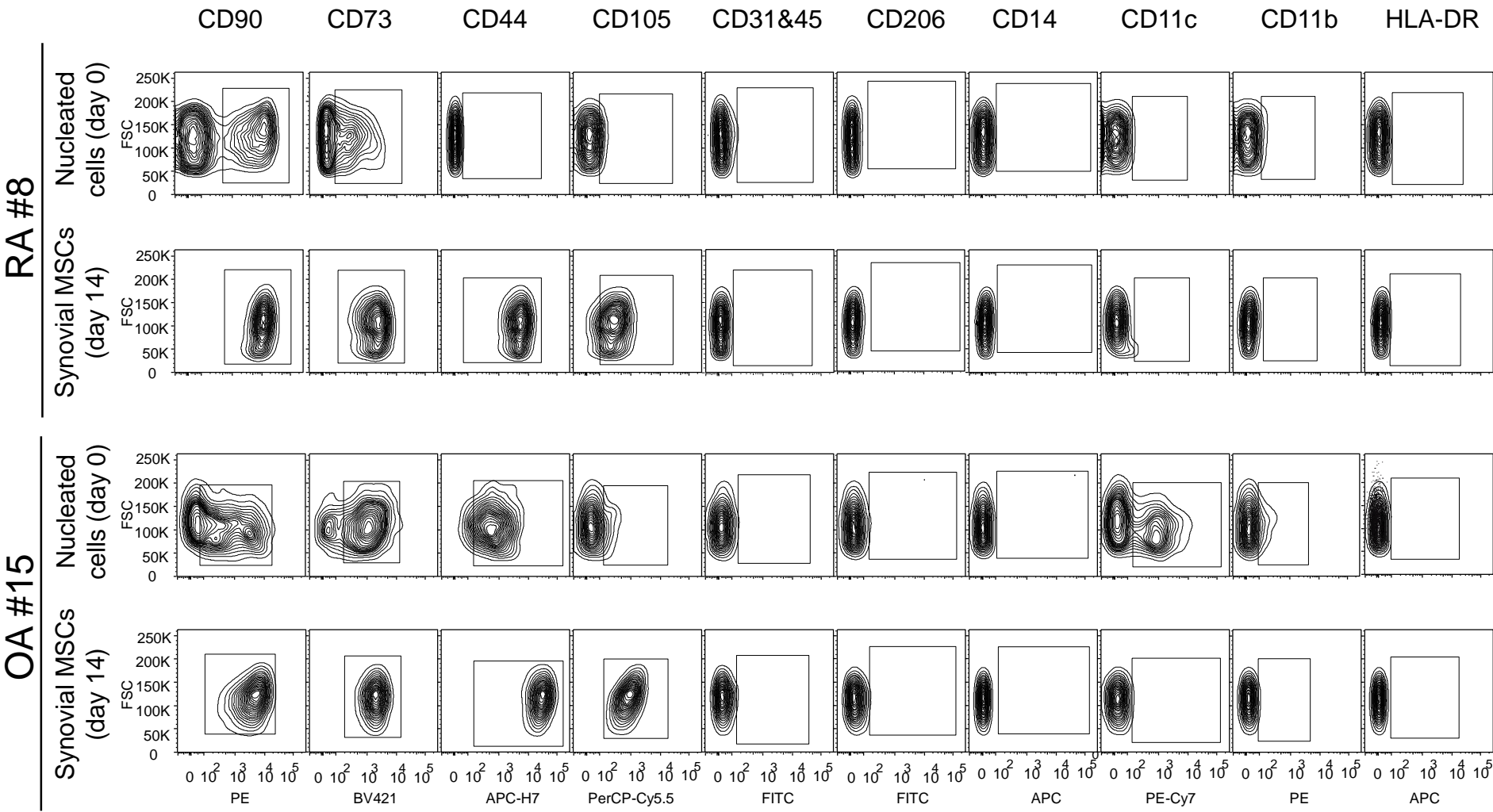

Supplement: Supplementary file 1 — Representative flow cytometry diagrams and isotype controls. (ZIP 836 kb) [file 13287_2017_572_MOESM1_ESM.zip › Kohno+RAvsOA+SCRT+Supplementary Figure 1+161007 0940.pdf]

Supplementary Figure

Isotype control

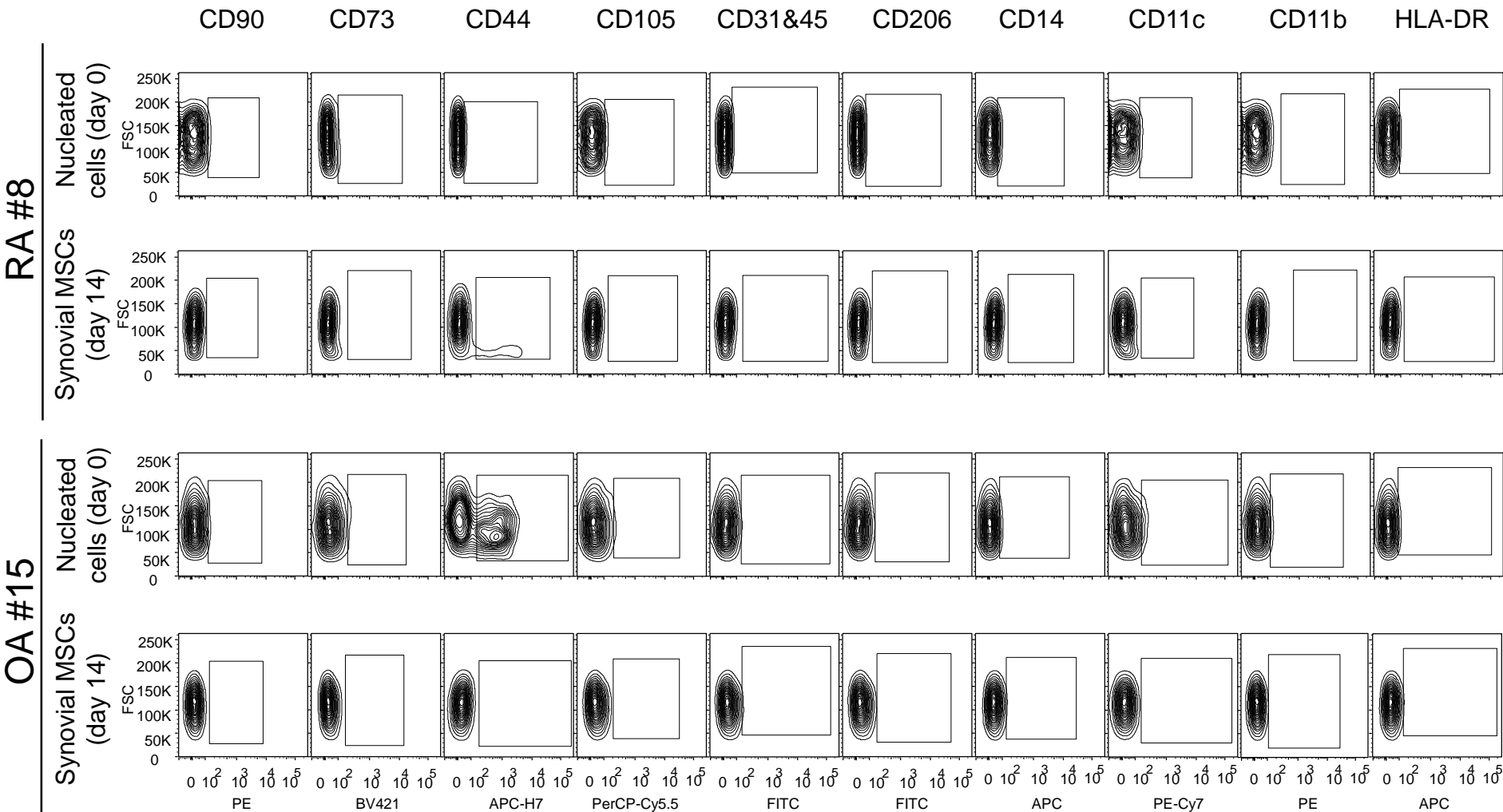

Supplement: Supplementary file 1 — Representative flow cytometry diagrams and isotype controls. (ZIP 836 kb) [file 13287_2017_572_MOESM1_ESM.zip › Kohno+RAvsOA+SCRT+Supplementary Figure2 +161007 0940.pdf]
