# Supplementary material for: Yields and chondrogenic potential of primary synovial mesenchymal stem cells are comparable between rheumatoid arthritis and osteoarthritis patients
Source: Stem Cell Res Ther. 2017 May 16;8:115. doi: 10.1186/s13287-017-0572-8 (PMC5434623; doi:10.1186/s13287-017-0572-8)
Supplement: Supplementary file 2 — Representative raw data for surface markers. Flow cytometric analyses of digested cells before plating (day 0) and expanded cells cultured for 14 days. Donor number of RA and OA patients shown. (DOCX 16 kb) [file 13287_2017_572_MOESM2_ESM.docx]

**Supplementary Table.** Positive rate of the surface epitopes (%).

|  |  | Nucleated cells  (day 0) | | |  | Synovial MSCs  (day 14) | | |
| --- | --- | --- | --- | --- | --- | --- | --- | --- |
|  |  | #7 | #8 | #1 |  | #7 | #8 | #1 |
|  | CD 90 | 34.2 | 40.6 | 41.7 |  | 99.8 | 99.4 | 99.9 |
|  | CD 73 | 30.9 | 29.5 | 57.6 |  | 99.8 | 98.9 | 100.0 |
|  | CD 44 | 99.3 | 0.0 | 37.7 |  | 99.8 | 99.5 | 99.4 |
|  | CD 105 | 8.2 | 7.8 | 1.5 |  | 97.5 | 76.8 | 96.8 |
| RA | CD 31& CD 45 | 94.5 | 3.2 | 24.5 |  | 2.1 | 0.1 | 0.5 |
|  | CD 206 | 1.7 | 1.2 | 0.5 |  | 2.6 | 0.1 | 0.7 |
|  | CD 14 | 0.8 | 0.1 | 0.0 |  | 0.0 | 0.3 | 0.0 |
|  | CD 11c | 13.8 | 0.0 | 3.6 |  | 1.8 | 1.8 | 1.0 |
|  | CD 11b | 33.3 | 1.4 | 3.1 |  | 3.3 | 0.2 | 0.1 |
|  | HLA-DR | 34.4 | 0.0 | 5.9 |  | 0.0 | 0.4 | 0.0 |
|  |  |  | | |  |  | | |
|  |  | Nucleated cells  (day 0) | | |  | Synovial MSCs  (day 14) | | |
|  |  | #16 | #15 | #10 |  | #16 | #15 | #10 |
|  | CD 90 | 42.1 | 63.4 | 32.1 |  | 99.6 | 99.7 | 99.7 |
|  | CD 73 | 68.4 | 82.8 | 40.2 |  | 99.7 | 99.9 | 99.7 |
|  | CD 44 | 36.8 | 97.5 | 0.0 |  | 98.6 | 100.0 | 99.7 |
|  | CD 105 | 5.3 | 9.4 | 14.6 |  | 90.9 | 99.7 | 62.7 |
| OA | CD 31& CD 45 | 15.8 | 2.6 | 16.4 |  | 0.2 | 4.7 | 0.1 |
|  | CD 206 | 0.4 | 2.9 | 4.4 |  | 0.4 | 6.6 | 0.2 |
|  | CD 14 | 0.0 | 0.0 | 2.8 |  | 0.0 | 0.0 | 0.3 |
|  | CD 11c | 7.1 | 42.3 | 3.5 |  | 0.9 | 2.0 | 1.0 |
|  | CD 11b | 0.5 | 9.9 | 12.4 |  | 0.3 | 0.7 | 0.3 |
|  | HLA-DR | 0.0 | 0.1 | 3.7 |  | 0.0 | 0.0 | 0.6 |
